# In Defence of Moral Pluralism and Compromise in Health Care Networks

**DOI:** 10.1007/s10728-018-0355-0

**Published:** 2018-03-29

**Authors:** Kasper Raus, Eric Mortier, Kristof Eeckloo

**Affiliations:** 1 Ghent University Hospital, Ghent University, Corneel Heymanslaan 10, 9000 Ghent, Belgium; 2 Faculty of Arts and Philosophy, Department of Philosophy and Moral Sciences, Ghent University, Ghent, Belgium; 3 Faculty of Medicine and Health Sciences, Ghent University, Ghent, Belgium; 4 Faculty of Medicine and Health Sciences, Department of Public Health, Ghent University, Ghent, Belgium

**Keywords:** Health care network, Collaboration, Consensus, Compromise, Pragmatism

## Abstract

The organisation of health care is rapidly changing. There is a trend to move away from individual health care institutions towards transmural integrated care and interorganizational collaboration in networks. However, within such collaboration and network there is often likely to be a pluralism of values as different health care institutions often have very different values. For this paper, we examine three different models of how we believe institutions can come to collaborate in networks, and thus reap the potential benefits of such collaboration, despite having different moral beliefs or values. A first way is the pragmatic way in which the different health care institutions avoid ethical reflection and focus on solutions. A second possible route is that of consensus where health care institutions base their collaboration on values that they all share. The third, and final, approach is that of compromise. Although moral compromise is often seen in a negative light, we argue that in many cases compromise might be necessary and ethically justified. In a final section, we will shift our focus from discussing various theoretical methods to allow collaboration to the potential content of consensus or compromise.

## Introduction

In the organisation of health care, there is a trend to move away from individual health care institutions towards transmural integrated care and interorganizational collaboration [28, 51]. The drivers of this trend towards integration and collaboration can be political, economic, ideological, pragmatic, etc. What exactly drives such collaboration between health care institutions differs significantly between health care systems, but the trend is undeniable. Across all systems, hopes are high for integrated care and health care networks (HCNs) which have been argued to potentially increase economic efficiency [10] or improve the quality of patient care [31].

There are also possible barriers. Forming HCNs requires at least some form of agreement on moral values or policies, as failing to do so could threaten the success or sustainability of the network when ethical conflicts arise [33]. Many, if not most, of the policy issues in an institutional health care context have a clear ethical or normative component. Examples may include policy on issues related to abortion or care at the end of life (e.g. withholding or withdrawing treatment and physician assisted dying). However, disagreements between institutions need not be limited to issues surrounding medical procedures and can involve, for example, policy on wearing and displaying religious symbols.

Moral agreement between health care institutions is far from self-evident as they often have very different missions, values and ethical norms. While health care systems have a legal framework setting the legal boundaries for collaboration, this is unlikely to do away with moral conflict as laws often leave room for interpretation and application. There exist a number of different reasonably acceptable views on the organisation and performance of health care and thus a ‘fact of pluralism’ [42].

For this paper, we want to examine three different models of how we believe institutions can come to collaborate in networks, and thus reap the potential benefits of such collaboration, despite having different moral beliefs or values. A first way to handle pluralism of values is the pragmatic way which involves restricting oneself to concrete disagreements and practical solutions. A second way is to build a common HCN perspective based on consensus between the network partners. The third way is to engage in moral compromise which involves mutual concessions.

We focus on agreement between institutions on values, principles, protocols and/or procedures on the meso-level of the network or the organizations of which the network consists. Although interesting, ethical issues at the macro-level (e.g. resource allocation within society at large) or micro-level (e.g. individual moral decisions by health care professionals) fall outside the scope of this paper.

## Health Care Networks and the Health Care Institutions that Make Up Such Networks

Before we proceed with our analysis, a few remarks are in order about our use of the concepts HCNs and health care institutions.

We use ‘health care institution’ as a broad concept which can refer to any public or private organization that provides health care or related services. This includes hospitals, but might also include such organizations as hospices, elderly care homes, primary care organizations, etc. As we focus on interorganisational collaboration, we do exclude parts of health care institutions such as particular departments. This broad approach accords well with the increasing focus on integrated care where the various health care services are not seen as completely separate, but are provided in a more coordinated and integrated way [34].

The emphasis on health care institutions is key to the approach taken in this paper. The primary focus lies on health care institutions *as institutions* rather than on a particular set of people that make up or represent those institutions. We will thus throughout the paper talk about health care institutions as being able to compromise or reach consensus. This implies we take the institution to have both some form of stable (moral) identity and a capacity for (moral) agency, although we acknowledge that this is the topic of considerable debate [17, 38, 49].

Dealing with this issue at length would go beyond the scope of this paper. We do however, believe our approach is justified. First, it allows us to focus on the formation of networks without overly complicating our analysis with forces and considerations that are at play *within* the individual organizations. We assume that in discussions about forming a HCN, individual institutions will come forward as a more or less single entity. Second, health care institutions are likely to meet several of what we consider essential criteria for identity or agency. In his important work on compromise, for example, Benjamin [6] distinguishes three structural conditions for identity: (1) a relatively stable set of values and beliefs, (2) verbal communication of those values and beliefs and (3) behaviour which corresponds with those values. Health care institutions meet these conditions as they arguably produce both policy documents pronouncing particular values and make policy decisions based on such values. As an example, some work has been done on Catholic hospital identity [43, 45].

Such health care institutions can collaborate and form HCNs, which we take to involve ‘*groups of three or more legally autonomous organizations that work together to achieve not only their own goals but also a collective goal*’ [39]. Compared to bilateral collaborations, networks are often amorphous and involve a complex set of interactions between the various members of that networks. They can thus take many different forms and be governed in a wide variety of ways [2, 39].

How and why such networks are formed can differ significantly from health system to health systems and significantly influences the necessity to cooperate. In some countries, governmental policy requires hospitals or other health care institutions to form networks. In Belgium, for example, the government is planning to require every hospital to become part of a larger hospital network, although the choice of network members is largely free. In other health systems formation of networks might not be legally required in which case networks will be formed on more voluntary basis. Even in such a system forming networks might still be a necessity in many cases for, for example, economic reasons. However, the point is that regardless of whether a particular network is formed for policy reasons, economic reasons or on voluntary basis, for the network to operate successfully the members of that network will have to find a way to find agreement. In this paper we will analyse three such ways.

Who the members of a particular network are, is also of relevance. A HCN involving only private for-profit institutions encounters different issues (e.g. competition issues) than do networks involving only public health care institutions (e.g. NHS hospitals in the UK) or networks involving both private and public institutions.

Bearing in mind this wide diversity of networks is important. When analysing the three models of collaboration, we argue that no model is inherently or automatically superior to the other models. Rather, which model for collaboration is best suited for a specific network depends on the health system one operates in and the composition of the network

## Pragmatism and ‘Muddling Through’

One evident way of dealing with differences of opinion within a network such as a HCN could be to not engage in a comprehensive reflection, but to instead deal with concrete issues as they arise. This could be linked to ‘muddling through’ [30]. It is possible that HCNs involving members with different moral opinions function successfully in absence of comprehensive agreement.

Theoretically, this seems to be linked to the philosophical tradition of Pragmatism. Historically the tradition of Pragmatism relates to the works of the classic American Pragmatists Charles Sanders Pierce, William James and John Dewey, but more contemporary philosophers such as Hillary Putnam and Richard Rorty can also be considered pragmatists. Central to the Pragmatist tradition is the idea that hypotheses and propositions are best assessed in terms of their practical consequences rather than on their truth or falsehood. Pragmatists note that focussing on falsity and truth has brought us no closer to resolving certain philosophical (or other) debates but has, quite to the contrary, fuelled unnecessary debate. For Pragmatist thinkers, ‘unless some practical difference would follow from one of the other side’s being correct, the dispute is idle’ [25].

The theory can be easily applied to the health care context, for example in debates concerning abortion. Central to the debate on abortion is the question of personhood, i.e. the question of whether or not embryos and foetuses are human persons who deserve the same protection as any other human being. A pragmatist thinker might note that despite centuries of debate, this discussion has not been resolved and propose shifting the debate towards the practical consequences of adopting a certain stance on personhood. Granting personhood to foetuses from the moment of conception in effect makes any abortion a form of murder, whereas denying personhood throughout the entire pregnancy might in effect justify abortion up until the time of delivery. Hospitals could then opt for the position that offers the best practical consequences, thereby abstracting from its truth or falsity. This theoretical model has also been applied to the ethics of ‘workarounds’, being pragmatic fixes to deal with moral problems in health care systems [7].

### In Practice

Pragmatism is a *result oriented approach* which involves examining whether particular solutions to a moral conflict would be acceptable to *all* members of the network. This might be the case without them agreeing on *why* this solution is acceptable. If so, further debate is idle as which side holds the truth makes no practical difference. The pragmatic approach thus assumes that comprehensive ethical debate is only relevant when there is disagreement on which way to go.

In practice, one could imagine institutions within a HCN having very different beliefs about the acceptability of physician-assisted dying (where this practice is legal). Whereas one institution believes it to be ethically justified another institution is adamantly opposed to it. Instead of debating the truth or falsity of these beliefs, these institutions might examine which position has the best practical consequences, thereby reaching the agreement that patients with a request for euthanasia are transferred to the institution more favourable towards euthanasia. The health care institution opposed to euthanasia could perhaps agree with this solution as they would not be required to change their stance on assisted suicide and euthanasia and would not have to perform it within their institutional walls. The other institution could agree on grounds that this arrangement would make sure that patients who want euthanasia (and fulfil all legal criteria) can indeed receive it. Also, if one considers the example of the network containing only public health care institutions which are part of the same larger health care system, it is clear that such an arrangement might not raise systemic ethical issues.

However, as we will mention below, such a solution might raise various other ethical issues (e.g. the issue of complicity). The approach is unlikely to be suited for networks containing health care institutions with fundamentally different moral beliefs (e.g. a network containing both religiously affiliated and non-religiously affiliated hospitals).

### Discussion

There are evident advantages to this pragmatist approach. It could allow network members to reach agreement more quickly than they would if they were attempting to reconcile fundamental moral beliefs. It might also allow network members to hold on to particular beliefs as the pragmatist approach allows institutions to adopt positions based on practical consequences and not truth. As such this might be a fruitful approach for particular networks.

However, there are considerable risks. First, there is the issue of whether it is conceivable or desirable for institution to abstract from the truth or falsity of their beliefs. What constitutes a good practical consequence is *determined* by one’s moral beliefs. If an institution believes euthanasia is morally wrong, they are likely to disapprove of a position that leads to euthanasia being performed more. They are likely to feel morally complicit if they do, even if euthanasia is not performed within their institutional walls. One might not only be morally responsible for what one does, but also for what one agrees to or approves of.

Second, which solution one prefers does not have to be the result of a reasonable reflective process, but might just as well be guided by mere intuition, emotion, circumstances, etc. There is thus no guarantee that the solution two or more institutions agree on is in fact ethically justified.

Third, the pragmatist method operates mainly in response to conflict. It is known from studies into the power relations in HCNs that a particular dominant group or partner might have the power to control the network agenda and, therefore, which conflicts surface and which stay hidden [1]. Hence a pragmatist method might play into the hand of the larger or more dominant partner within a HCN.

## Moral Consensus

Another way to deal with pluralism of value within HCNs is to look for (overlapping) consensus. Between HCN members, there might be underlying agreements which could serve as a basis for collaboration. The most famous theoretical model for consensus in philosophy and ethics is Rawls’ model of ‘overlapping consensus’ [41] as brought forward in his *A Theory of Justice* [40], but perhaps more extensively in his book *Political Liberalism* [42]. Although mainly aimed at organizing political and social life, Rawls’ thinking could just as well be applied to HCNs.

### In Practice

In practice, the consensus approach amounts to a search for those values on which consensus exists. In that respect it is broader than the pragmatic approach which stops at agreement on solutions.

Such an approach could be considered feasible for HCNs, especially where the members of that network are likely to share moral beliefs. One could imagine a HCN consisting of Catholic inspired health care institutions who are likely to find common ground in their religious affiliation. However, consensus can just as well be reached across ideologies, for example by referring to the goals of medicine. An example was given by the late pope John Paul II who said:

I hope that Catholic health care institutions and public health care institutions may be able to collaborate effectively, united by the common desire to serve the human person, especially, the weakest and those who, in fact, are not socially insured [29].

In case there is no immediate consensus, there is the hope that such consensus might gradually grow. In the context of HCNs, some commentators argue that ‘the process of reaching consensus regarding values is troublesome and largely unfolds through the opportunity of working side-by-side’ [44: 314]. There is thus a possibility that a pragmatic approach to HCNs could evolve into a consensus approach.

### Discussion

Starting from a consensus has the clear advantage that the result of the consensus is acceptable to all parties. Within health care, certain values (e.g. patient centrality) are prime candidates as starting point for collaboration because they are widely shared among health care institutions and are often also enshrined within the legal framework. Reaching consensus might also bring institutions closer together by requiring them to focus on what binds them rather than what divides or distinguishes them. A consensus model allows institutions to stay true to their perceived identity by creating an area where institutions collaborate based on shared and agreed upon rules, values or principles.

Despite the obvious benefits, consensus might also have its drawbacks. For some commentators a true consensus is practically unfeasible, but also theoretically void [14, 15, 36]. In view of pluralism, the only thing there will be agreement on is likely to be highly abstract and/or vague. Thus, when consensus is reached, one might wonder how deep or superficial it is. Foster et al. [16] found substantial overlap with the codes of ethics of over 500 companies, with some even being identical. The authors suggest that many companies simply express ethical values that are common or popular. A similar process could take place in mission statements or codes of ethics of health care institutions. For example, while hospitals might agree, as stated by John Paul II, on a ‘desire to serve the human person’, they might differ in their interpretation of what this means in practice. If this is the case, the value of a general consensus is severely diminished as it is still likely to result in conflict in practice.

## Moral Compromise

Compromising is another way of dealing with a plurality of values. The seemingly simple concept of compromise has been shown to be a rich and complex notion [6, 19]. Initially, there seem to be three ways in which the concept of compromise is used: (1) compromise as betrayal; (2) compromise as a process; and (3) compromise as an outcome [6].

Compromise is often used to indicate betrayal. For example, we can say of a policeman caught stealing that his/her professional and moral integrity is compromised. In the context of moral conflict, compromise has indeed been argued to be particularly problematic [11, 24]. One’s moral values or moral beliefs are claimed to be unsuited for negotiations or concessions. Although we acknowledge these issues, we will argue that classic barriers to moral compromise are not *necessarily* insurmountable. We will thus use the concept in a non-evaluative way in this paper. Although there, undoubtedly, are bad compromises, this is due to the nature of these compromises rather than the mere fact that they are compromises.

For this paper, we understand compromise to refer to the process of resolving agreement through negotiation and normative concessions or, as argued by Benjamin, by ‘splitting the difference’ [6]. The outcome of that process could also be labelled a compromise but, unlike the process, the outcome does not necessarily involve mutual concessions. For example, two parties in disagreement might agree to have their disagreement resolved through a decision by a third party. In this case the compromise lies in the fact that both parties agree beforehand to accept the decision by the third party, even if the outcome of the decision is one-sided. Therefore, we believe that what truly distinguishes compromise from consensus or pragmatic decision-making is the process rather than the outcome.

When applied to HCNs, compromise is a third strategy for handling a plurality of values amongst the network members. This strategy differs in one important respect from the previous two strategies which both start from agreement. In pragmatism there is agreement on solutions and in consensus there is agreement on basic principles or values. This, in both strategies, forms a natural starting point and incentive for network collaboration. Compromise, by contrast, starts with disagreement and involves *reaching* some degree of agreement. What can incentivize network members to nevertheless contemplate compromise as a strategy for collaboration? We believe in some cases collaboration between health care institutions can be required and/or desirable, even while there is insufficient agreement for a pragmatic solution or consensus. In such a case, as remarked by Benjamin [6] compromise can be considered as a strategy for ‘preserving continuing, cooperative relationships’, which might be beneficial all things considered. It is increasingly being acknowledged that integrated care and interorganisational collaboration can greatly benefit patients [18], communities and even society at large [34]. Health care institutions arguably have an obligation to further not only their own interests, but also those of patients, the community they operate in and the society of which they are part. In such cases if there is consensus between institutions on cooperation as a beneficial *goal*, but insufficient agreement for a consensus approach as a *means* to achieve that goal, this could incentivize members to consider compromise as a means to nevertheless achieve cooperation.

### Barriers to Compromise in Health Care Networks

We acknowledge that compromise as a strategy for collaboration nevertheless faces considerable barriers of which we will discuss the two main ones.

- Ethical values as constitutive of (corporate) identity and integrity

One of the reasons why compromise on moral beliefs, values or norms is difficult is that these are often *constitutive* of one’s identity [13]. Hence one does not subscribe to a certain ideology *and* have certain values, but rather one has particular values *because* one subscribes to a particular ideology. Compromising on certain deeply felt ethical beliefs, values and norms could then be felt as a betrayal of one’s identity and thus as a loss of moral integrity. Moral integrity for *individuals* is widely recognized in the health care context and forms the justification for conscientious objects whereby a health care professional refuses to assist or perform in a certain medical act that is irreconcilable with one’s moral beliefs.

However, for institutions the issue of moral integrity is less often discussed. Corporate (or institutional) identity has often been linked to branding and marketing rather than moral integrity [4]. If so, an incoherent identity might for a health care institution be more of an image problem than an ethical issue of integrity. However, we think that for institutions identity should be seen as more than a mere branding issue. We have argued above that there are reasons for considering institutions to have moral identity and agency. Moreover, various institutions do highly value their identity and do consider some collaborations to pose threats to that identity. Witness to this fact are the *Ethical and Religious Directives for Catholic Health Care Services* issued by the US Conference of Catholic Bishops as recommendations to Catholic healthcare institutions. They remark that:

On the other hand, new partnerships can pose serious challenges to the viability of the identity of Catholic health care institutions and services, and their ability to implement these Directives in a consistent way, especially when partnerships are formed with those who do not share Catholic moral principles [46].

Their recommendation, subsequently, is to only collaborate with non-Catholic health care institutions when collaboration with a Catholic health care institution is not possible. Thus when there is, relatively, free choice of partners to collaborate with, this very choice might already involve some form of compromise.

Similar considerations might be at work with other ideology related health care institutions such as, for example, Jewish hospitals [22]. For example, in 2015 when various Jewish hospitals were grouped in a single entity, the CEO of that entity subsequently proclaimed that in the future he would not compromise on the Jewish character of the hospitals in the group and stated that*: ‘*Our culture is absolutely non-negotiable. Our forefathers built these institutions. They are our heritage’ [3].

(b) Non-negotiability of moral claims

Health care institutions might not be willing to compromise on moral beliefs or values because they believe them to be non-negotiable by nature. Some hospitals, for example, may consider life to be absolutely sacred and they might therefore have a complete prohibition on any form of abortion or shortening of life. Any deviation from that prohibition is perceived as a denial of the sanctity of life principle and they might therefore not be willing to collaborate with institutions that do allow these practices to be performed.

The principle of rationality—often considered to be an essential requirement of ethics—requires that if one holds a value to be true or applicable to a certain situation, one ought to hold this value true or applicable in *all* relevantly similar situations. Absolute moral rules leave little wiggle room. Opposing moral values, principles or ideologies might thus stand off without there being a continuum between them where both parties might meet.

### In Defence of Moral Compromise

Together with several commentators [6, 26, 27, 37, 48] we argue that the barriers discussed above need not necessarily be insurmountable.

First, compromise is not so irreconcilable with identity or integrity as is often assumed. Identity is not a fixed and timeless concept, but an ever evolving entity. It has been argued extensively that achieving a complete and fully integrated set of moral values and principles is not possible [6, 48]. Constructing and maintaining identity should be seen as a continuous process. Health care institutions might be required to constantly rethink or adjust their identity in view of important changes in the health care. Reflecting, dialoguing and compromising with other institutions could and should be seen essential parts of that process. Understood in this way, compromising is a formative process that might build or strengthen one’s identity instead of destroying it. Also, within a HCN a new, higher level, identity might be formed.

Second, as has been noticed by many commentators, a willingness to compromise can in itself be a moral principle that one avows [6, 26, 27]. It can show respect to the moral position of others and a willingness to ‘get things done’. In many cases in the health context a decision is needed and unavoidable, providing an ideal condition for compromises to become possible. As such, compromising could actually be evidence of one’s moral integrity.

Third, it was mentioned that many ideologies or ethical theories have non-negotiable ethical claims. To fully discuss this problem would go beyond the scope of this paper. However, we would like to touch upon one particular issue. We argue that it is possible to make a distinction between the question ‘What is the best principle/value/rule/solution?’ and ‘What is the best principle/value/rule/solution in this particular context, at this particular time within this particular community?’. The former question is the truly non-negotiable one, whereas the latter might be the topic of debate and varies from context to context or from time to time. Joseph Heath once made the parallel that if your primary preference is to fly to Hawaii and you know you only have fuel to get 90% of the way, the best option is not to travel 90% of the distance, but to choose an entirely different destination that is reachable [23]. Health care institutions might thus be able to compromise on a particular policy while, at the same time, holding on to their fundamental moral beliefs and principles [26].

### In Practice

As regards the use of compromise in practice, we will examine two process-oriented approaches.

#### Habermasian Discourse Ethics

A known model for compromise is that of Discourse Ethics defended by, for example, Habermas [20, 21]. This theory shifts focus from moral beliefs or principles to rational discourse and debate. The philosophical reasoning is that ethical principles or truths cannot be discovered by individuals on themselves, but only through communicative rationality and discussion with others. Deliberation and discussion is not what is needed to arrive at the right answer, but is, by contrast, what *makes the answer right*. Off course, in order to function properly, there are several conditions that have to be met. For example, all parties must communicate in all honesty and must be willing to reach a solution. For Habermas true deliberation has to imply a willingness to alter one’s own beliefs throughout deliberation. Whereas some ethical models see others as people that have to be convinced and brought to one’s own perspective, Habermas sees others as potential partners in a joint quest for the truth. The focus lies on the process rather than the result and as such, it might be said to be radically opposite to the pragmatic approach discussed above.

The Habermasian method has been applied in practice. Walker and Lovat [50] have applied the theory to develop a model for ‘dialogic consensus in clinical decision-making’. Another method akin to discourse ethic is ‘moral case deliberation’ [35]. Here a group is faced with an ethical conflict and attempts to resolve this by going through a set of fixed phases of explanation, discussion and deliberation.

When applied to health care *networking*, this theory implies that network partners could attempt to resolve differences through agreed upon process. Potential HCN members might come to agree on how ethical issues should be discussed and reflected on and might agree to accept whatever results from that discussion. Imagine, for example, various health care institutions in a network disagreeing on how members of that network should respond to certain patient requests (e.g. requests for abortion). Although it might be impossible for them to agree on principle particular outcome, they might perhaps agree on a particular process for resolving the difference. This could, for example, involve the creation of a network wide ethics committee that contains representatives of the various institutions and certain procedures for that ethics committee. Richard Huxtable has convincingly argued for the possibility and the advantages of resolving moral conflicts through the use of ethics committees [26, 27].

#### (Wide) Reflective Equilibrium

A second model of moral compromise for HCNs could be the Rawlsian idea of a (wide) reflective equilibrium [12, 42] where the HCN attempts to arrive at a coherent and justified set of moral beliefs. The goal of reflective equilibrium is to create a balance between one’s considered moral judgements in particular cases and the principles one aims to uphold. If these are mutually inconsistent, for example when the person fails to apply a moral principle in all similar cases, she can either change her considered judgement or the principle she adheres to. Hence reflective equilibrium is a fundamentally deliberative process whereby a person goes back and forth between her judgements and her avowed moral principles, until coherence is reached.

There have been attempts to discuss how such a theory would work in practice [47], including the practice of health care delivery. Reflective equilibrium requires institutions or HCNs to engage with the values or principles they support to see whether they apply these principles consistently and whether they are coherent with other values and principles they avow. If principles are found not to be applied consistently, equilibrium must be restored by changing the institution’s or network’s stance in particular cases or by changing the principle. Likewise when two values are incoherent, this requires the changing or adjusting one of these values. If one changes the principle, one has to consider the consequence of that change and whether this effects the coherence of the new principles with other principles. Hence searching for an equilibrium is a continuous project.

To indicate how reflective equilibrium might work, we provide a hypothetical example. Imagine, for example, a network that includes multiple hospitals and in which two of those hospitals uphold two principles. The first hospital values the principle: *within our HCN, patients should always be given the best possible care*. The second hospital avows the principle: *competent patients must always be able to make their own autonomous choices regarding their own care*. Within the network context, these two principles might be mutually exclusive. Imagine one health care institution within that network (institution A) specialises in a specific type of heart surgery and in quality audits has markedly better performance scores. If some patients in need of that heart surgery want to have this surgery done in institution B within that network (e.g. because this hospital is nearer to their residence), this can cause a problem of coherence. Either the network allows these patients to have the surgery done in institution B (thereby failing to provide the best possible care) or they require these patients to have the surgery done in institution A (thereby limiting these patients’ autonomous choice). Following reflective equilibrium the network has to address this incoherence. For example, they might attempt to address the quality differences and try to make sure the specific heart surgery is done with the same standard of care in all institutions (e.g. by buying equipment or providing additional training for the surgeons of the other institutions). In such a scenario patients could be able to choose *and* receive the best care. However, in practice doing away with this quality gap is far from easy and might require a significant amount of resources. Second, the network might choose to adopt the following principle: *within our HCN, patients should always be given the best possible care that is respectful of their autonomous choices*. This would alleviate the inconsistency between the two institutions’ principles, as patients having the surgery done in institution B would not receive the best possible care, but they would receive the best possible care considering their autonomous choice of not going to institution A. As such, this principle could be a good compromise for both institutions as it combines and balances quality concerns (particularly relevant for one institution) and autonomy (particularly relevant for another institution).

An advantage is that reflective equilibrium starts from network members’ own values and moral beliefs, but then tests these, primarily, for internal coherence and consistency. Instead of choosing one of the mutually exclusive principles, this equilibrium approach requires institutions to find a way to make these principles consistent with each other (e.g. by slightly altering one’s avowed values as an institution). Reflective equilibrium does not lead to moral relativism but should, on the contrary, lead to a more coherent set of moral judgements and a more consistent application of moral principles.

### Discussion

As argued above, we focus on compromise as a process. Essential is that in both models discussed above, institutions do not alter their beliefs in response to another party with an opposing view (which might be considered ‘caving in’) but rather as a result of a mutually undertaken deliberation process.

However, there are red lights to bear in mind. First, as this method does not require a definite choice, it might also be perceived as a way to delay ethical choice or even to avoid making any ethical choice at all. If the method is used in this way, it falls victim to the same critique that was brought against pragmatism, namely that the method resolves ethical conflict by failing to address the ethical issue. Second, the method does not stipulate who should take part in this discourse. Imagine a case where all hospitals in a large geographical area form a HCN. This would have important ramifications for people living in that area (e.g. regarding access to health care). Hence one might wonder whether, considering their interest, these people should play a role in this discourse as stakeholder and what that role should be. Third, within health care various different ‘discourses’ exist (e.g. an economical discourse, an ideological discourse, etc.). When forming a HCN, a dominant partner in that network might control the discourse being used, thereby exerting considerable power over the network and its various partners [6]. Hence what should be an open debate, can be hijacked by one of the network partners.

## Consensus and Compromise: The Search for a Common Ground

In the previous sections we have examined models which constitute the various different *approaches* to achieve consensus or compromise. In doing so, we have somewhat abstracted from the question what the *content* of moral consensus could be. In this final section, we will therefore focus on this issue. Imagine two or more health care institutions are intent on forming a partnership or HCN and are trying to work through their moral differences. They might aim at resolving moral differences one dilemma at a time or they might try to design a more comprehensive (normative) framework.

Institutions might attempt to resolve arising ethical conflicts on a case by case basis, focussing on particular *solutions* on which everyone agrees. We have argued that although such an approach might be tempting from a practical point of view, it often comes at an ethical cost. Ad hoc decisions might result in a net loss of overall fairness or justice within the HCN. In many cases to guarantee justice, some form of consensus or compromise is necessary. Concerning the content of consensus or compromise, we will examine various candidates that range from (1) broad to specific and from (2) vague to applicable.

A first obvious candidate are institutional moral values. Most, if not all, HCNs have particular avowed values such as, for example, patient centeredness and integrity. Such values can be argued to be both *broad* and *vague*. They are broad as they cover a great deal of cases or practices, which might make them an ideal candidate for a consensus approach as members of a HCN might be able to avow the same broad value. However, there may be some concerns regarding their immediate usefulness and applicability in resolving potential conflicts. Values tend to be vague and their application in concrete situations often requires substantial interpretation. This is, of course a potential cause of conflicts within or between the partners of the HCNs, in which case not much is gained by avowing shared values.

HCNs who agree on particular values could, of course, attempt to remedy the vagueness by, for example, translating certain values into particular goals or commitments. The value of ‘best quality for all patients’ could be translated into very concrete health care goals (e.g. a particular reduction of patient readmissions or an increase in patient reported quality of care). The advantage of such commitments is that they are concrete and it can be the topic of empirical study which measures best help to achieve these goals. The past decades monitoring and evaluating hospital performances and quality of care have been high on the agenda. Evidence based medicine has been praised as an important tool in the ethical management of hospitals [8] and as an important tool in bioethical decision-making [9]. Of course, translating values into concrete goals or commitments in no way guarantees consensus or compromise. First, health care institutions might disagree on what the specific goals should be. Second, there can still be disagreement on the correct way to achieve such concrete goals or commitments.

Formal ethical rules or an ethical code hold the opposite end of the spectrum as they are *applicable* and *specific*. Ethical rules are drafted to provide clear and unequivocal guidance for action in particular situations, thereby providing clarity. HCNs are often formalised in contracts or in the formulation of a joint mission statement. Ethical rules and other arrangements could be part of that contract or missions statement. However, as with values, their advantage in applicability may play against them when it concerns broad and flexible use of the moral framework. Rules are specific to the situation to which they apply and the act they proscribe, which may be problematic. First, no set of rules is likely to cover all cases or dilemmas that may arise. For such cases, HCNs may still require a more broad moral framework to fall back on. Second, rules are often straightforward whereas actual cases are complex. Being overly attached to particular rules can amount to a formalistic application of rules devoid of any ethical reflection. For this reason, MacIntyre famously referred to regulation and rules as a mere ‘substitute for morality’ [32].

Principles seem to combine the best of both worlds as they are drafted to be both *broad* and *applicable*. The general idea is that by reverting to principles, one has a framework that can give clear guidance, while being broad enough to remain attentive to complex cases. Rules can only be followed or broken, but principles can be balanced. The most well-known theory within biomedical ethics is of course Beauchamp and Childress’ ‘Principlism’ [5]. Much, however, depends on how such principles are formulated. When formulated vaguely, they are akin to values and suffer from the same problems. If formulated too specifically, they are akin to rules, with all associated problems.

However, any set of principles needs a theoretical framework concerning how principles should be applied, how they should be balanced or prioritised. Beauchamp and Childress’ principlist framework is famous for claiming that no principle has automatic priority over another principle. However, when principles conflict, a decision should be made. If there is ad-hoc or unsystematic balancing of moral principles, this can clearly result in injustice or unfairness. Hence, HCNs coming to a consensus or compromise on particular principles, should also have a method for operating these principles.

As was discussed above, another possibility is that within a HCN there is agreement on formal ethical procedures or processes. This outcome is somewhat the odd one out as it resists definite classification in the broad-specific and vague-applicable range given above. Procedures might be considered broad as a great variety of different dilemmas can be inputted into this process, but they also describe a particularly specific process. To some degree processes are also vague as they describe the process rather than the outcome, but they generate solutions to ethical issues and are thus applicable. As such, processes might cover the entire spectrum. Focussing on the process implies a commitment to profound and critical ethical reflection and it requires one to engage with one’s own beliefs or those of others in an attempt to resolve normative conflict. Of course, there may also be potential problems. Discussing and deliberating is time-consuming and thus not always workable. Procedures and protocols need some sort of stopping point at which case the discussion results in an outcome. Also, for members of a HCN providing medical care this might not provide the necessary guidance or security in their day-to-day work. Hence amidst the deliberation and discussion, practical outcome must also be guaranteed.

## Conclusions

We have argued it is becoming more and more common for health care institutions to collaborate in HCNs. These collaborations can be the result of governmental policy or other considerations. Whatever the reason might be, these networks will often comprise of institutions with a—to some degree—different moral identity. In this paper three ways to deal with such differences in identity and moral beliefs or values were examined. Which way is to be preferred, depends on the circumstances and the context in which these networks are formed.

A first way is the way of pragmatism or ‘muddling through’ which involves dealing with dilemmas by focussing on *solutions* on which agreement exists. For some networks, this way might be appealing. However, there is a risk that this leads to avoiding ethical debate or reflection. Another way is to use a consensus model when building HCNs, which involves focussing on those values and principles on which consensus exists. For networks involving institutions with overlapping moral beliefs, this approach might be useful. The risk with such a model is that the content of consensus might, in cases of moral debate, by very vague and thin. To what extent this consensus can thus always serve as a stable base for interorganizational collaboration is questionable. A third more controversial, route is through moral compromise which involves mutual concessions. Although moral claims are often argued to be non-negotiable, we have argued that compromise might nevertheless be reached through a deliberative process involving willing partners.

In sum, there are various possible grounds for consensus and compromise. None of these form the universal gold standard. It is clear, however, that they have their particular characteristics and disadvantages. Values, rules, codes of ethics, principles and procedures do not automatically and in themselves guarantee justice and fairness. If health care institutions come together to form a HCN, they will have to come to some degree of agreement on values, rules, principles or procedures, but also to take action to make them work in practice. Values may need more concrete content, whereas rules need a mechanism that allows for flexibility. Principles need a method for applying and balancing them.
